# Supplementary material for: Direct observation of topological surface-state arcs in photonic metamaterials
Source: Nat Commun. 2017 Jul 21;8:97. doi: 10.1038/s41467-017-00134-1 (PMC5522455; doi:10.1038/s41467-017-00134-1)
Supplement: Supplementary file 1 — Supplementary Information [file 41467_2017_134_MOESM1_ESM.pdf]

File name: Supplementary Information
Description: Supplementary Figures, Supplementary Note and Supplementary References

File name: Peer Review File
Description:

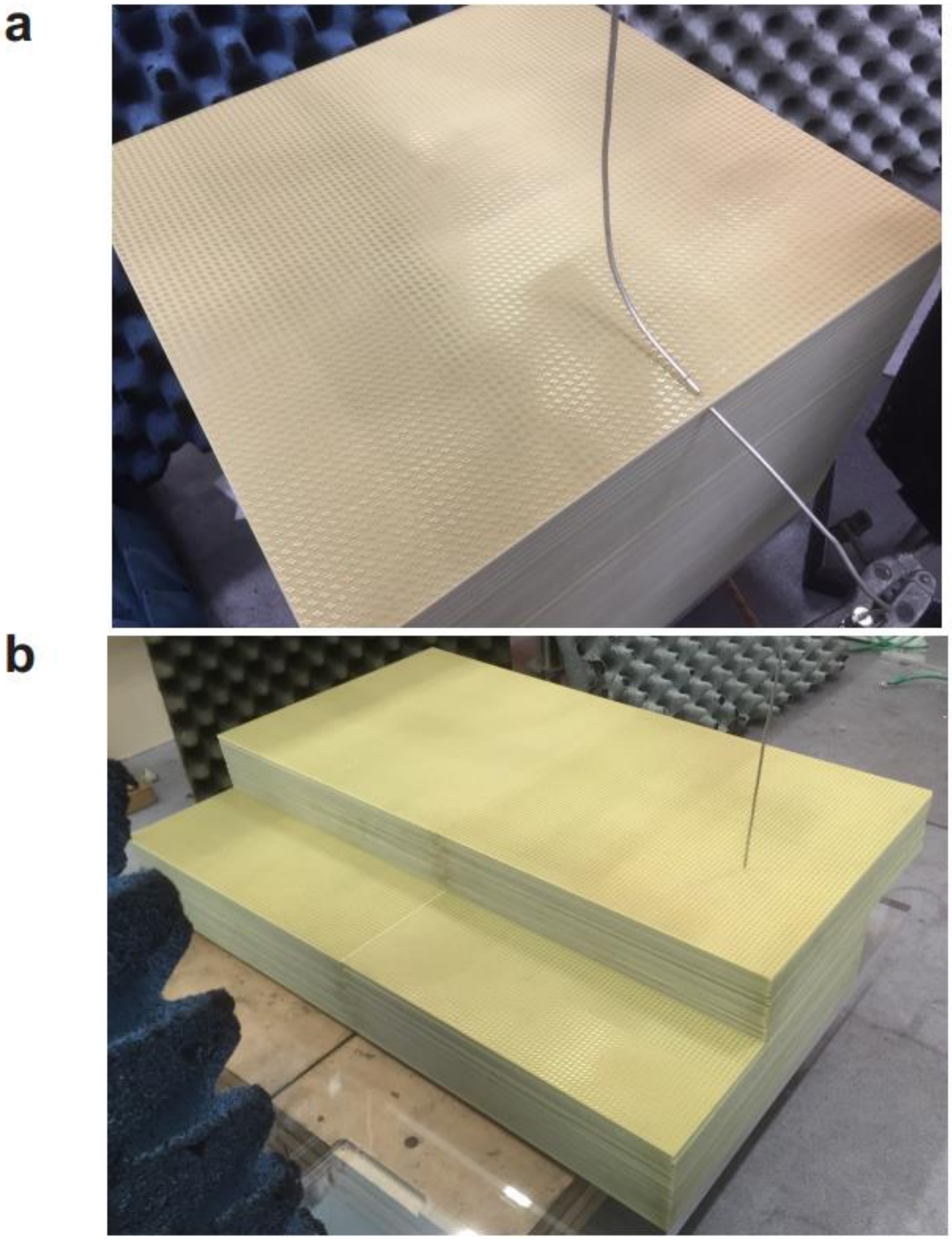

Supplementary Figure 1. Different sample configurations and experiment setups. (**a**), A bulky material is built with 75 periods along the *z*-direction, which was used to measure the top and side topological surface-state arcs (TSA) by scanning the field distribution on both surfaces. In the figure there are two probes, one of them acts as the source and the other one is the probe. (**b**), A step configuration is built to test the non-

triviality of surface waves propagation. Four blocks are used to construct the step. The step width, height and length are 104 mm (both for upper and lower surfaces), 60 mm and 600 mm, respectively.

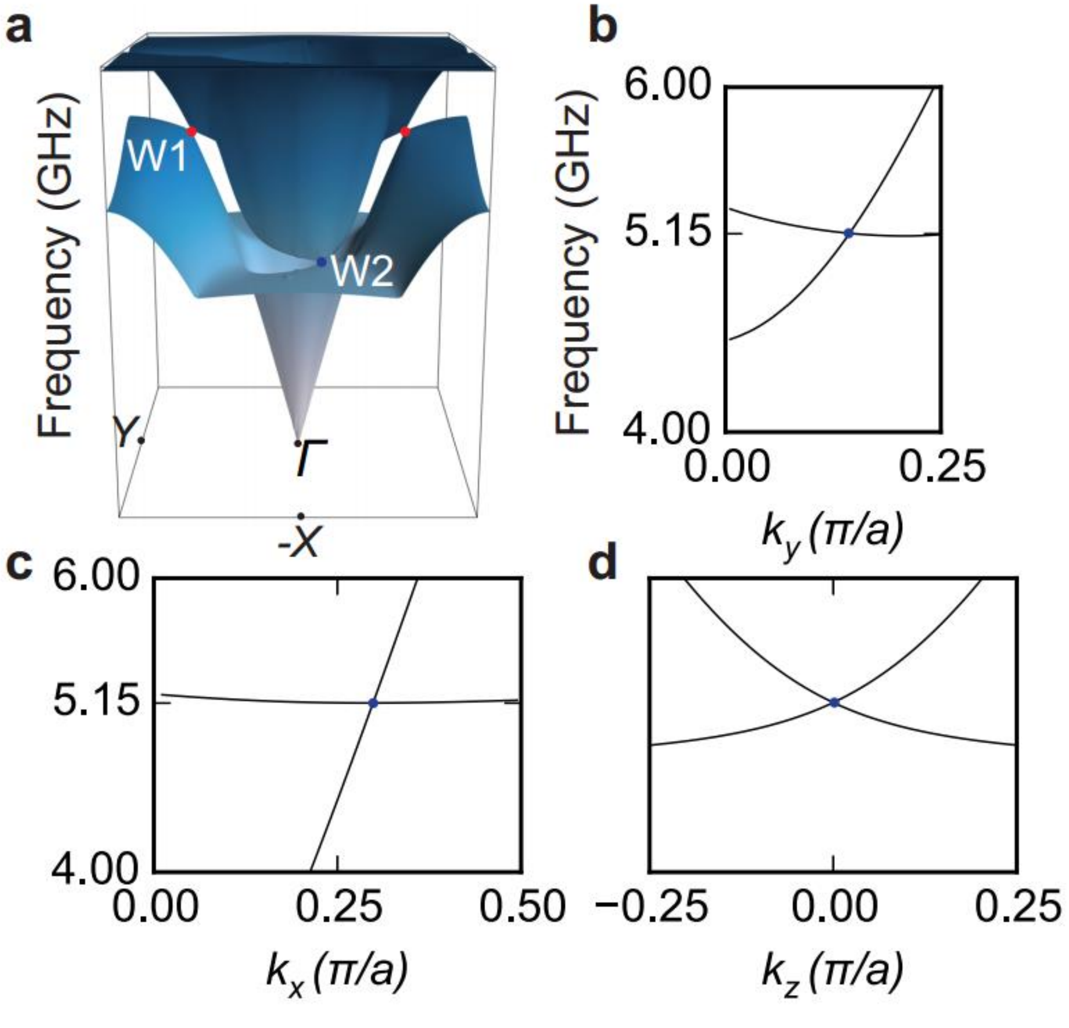

Supplementary Figure 2. Dispersion relations simulated from realistic structures by CST Microwave Studio. (**a**)**,** Energy dispersion on k_x - k_y plane with k_z = 0, the Weyl points are marked with red and blue spheres (one W2 is blocked). (**b**)**,** (**c**) and (**d**) show linear dispersions along three orthogonal directions in the vicinity of W2. From **a**, we can see four Weyl points similar to the prediction by the effective medium model in Fig. 1c

of the main text. Due to bi-anisotropic effect, those Weyl points in the real structures slightly shift away from the high symmetry axis. However, they cannot annihilate each other because the chiral partners (W1 and W2) are located at different frequency. Thus from this perspective, they are very robust against weak perturbations. In **c**, one band along k_x direction is almost flat and the group velocity approaches to zero. This is due to the strong resonance induced by chiral helixes.

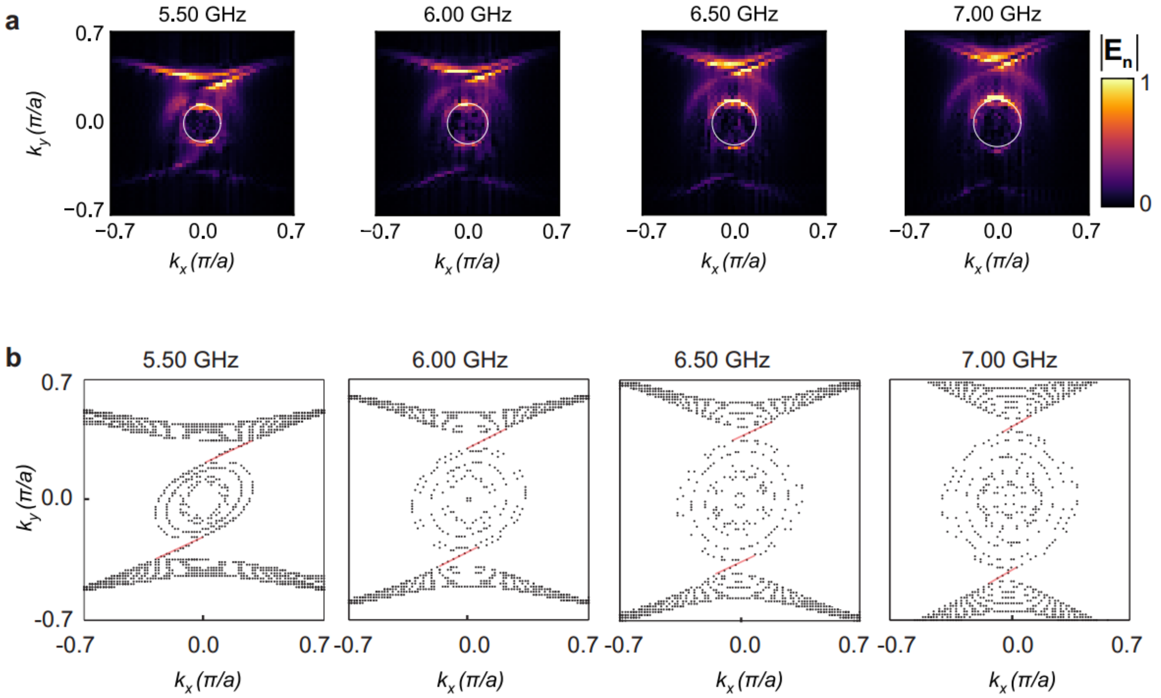

Supplementary Figure 3. Experimental and simulated equi-frequency contours with respect to different frequencies scanned on the top surface. (**a**) shows the experimental results with the polarization configuration that is a *z*-polarized source detected by a *z*-polarized probe. In all panels, the normal components of the electric fields are normalized to the same reference value. White circles represent the light cone. Recalling Fig .1g in the main text, topological surface-state arcs (TSA) serve to bridge the nontrivial gap. From 5.50 GHz to 7.00 GHz, the experiment results show strong

resemblance with simulation results shown in (**b**), which are simulated in CST using supercell with length of 20 unit cells along the *z*-direction. Red line segments highlight the TSA. **a** and **b** panels share the corresponding *y* axis, respectively.

Supplementary Note 1. Effectively modelled Hamiltonian

The realistic structure consists of a chiral layer (metallic springs oriented along *x* direction) and a hyperbolic layer (continuous metallic wires along the *y* direction). By considering the motion of electrons driven by external electromagnetic field on those metallic components[1,2], a Hermitian Hamiltonian can be obtained to describe the dispersive system,

$$H\boldsymbol{\psi} = N^{-1/2}MN^{-1/2}\boldsymbol{\psi} = E\boldsymbol{\psi} \tag{1}$$

where M and N are the matrices describing the dispersive properties of the effective model,

$$M = \begin{bmatrix} 0 & 0 & 0 & 0 & k_z & -k_y & 0 & -i\dfrac{\alpha}{\beta} & 0 \\ 0 & 0 & 0 & -k_z & 0 & k_x & 0 & 0 & -i\omega_p \\ 0 & 0 & 0 & k_y & -k_x & 0 & 0 & 0 & 0 \\ 0 & -k_z & k_y & 0 & 0 & 0 & 0 & 0 & 0 \\ k_z & 0 & -k_x & 0 & 0 & 0 & 0 & 0 & 0 \\ -k_y & k_x & 0 & 0 & 0 & 0 & 0 & 0 & 0 \\ 0 & 0 & 0 & 0 & 0 & 0 & 0 & i\omega_0^2\dfrac{1}{l\beta} & 0 \\ i\dfrac{\alpha}{\beta} & 0 & 0 & 0 & 0 & 0 & -i\omega_0^2\dfrac{1}{l\beta} & 0 & 0 \\ 0 & i\omega_p & 0 & 0 & 0 & 0 & 0 & 0 & 0 \end{bmatrix} \tag{2}$$

$$N=\begin{bmatrix} \varepsilon_x & 0 & 0 & 0 & 0 & 0 & 0 & 0 & 0 \\ 0 & 1 & 0 & 0 & 0 & 0 & 0 & 0 & 0 \\ 0 & 0 & \varepsilon_z & 0 & 0 & 0 & 0 & 0 & 0 \\ 0 & 0 & 0 & 1 & 0 & 0 & 0 & 1 & 0 \\ 0 & 0 & 0 & 0 & 1 & 0 & 0 & 0 & 0 \\ 0 & 0 & 0 & 0 & 0 & 1 & 0 & 0 & 0 \\ 0 & 0 & 0 & 0 & 0 & 0 & \omega_0^2 \frac{A}{l^2\beta} & 0 & 0 \\ 0 & 0 & 0 & 1 & 0 & 0 & 0 & \frac{1}{\beta A} & 0 \\ 0 & 0 & 0 & 0 & 0 & 0 & 0 & 0 & 1 \end{bmatrix} \quad (3)$$

In the model, we neglect the ohmic loss and the interactions between intra and inter layers. The slight bi-anisotropic effect mentioned in main text has also been neglected in the effective model for sake of simplicity, as bi-anisotropy merely shifts the Weyl points around in the momentum space and cannot gap them. ω_p indicates the effective plasma frequency for hyperbolic layers. l and A indicate effective length and area of the metallic helix, respectively. L is the effective inductance. The resonance of chiral layers is controlled by ω_0. Difference in ε_x and ε_z is due to the anisotropy induced by cross structures in hyperbolic layers. Spatial non-local effect is considered through introducing an even order (keeping time-reversal symmetry) k_y fraction in the effective plasma frequency,

$$\omega_p = 1 + \frac{ak_y^2}{1+bk_y^2} \quad (4)$$

Energy dispersion and surface states on the top surface are calculated as shown in Fig. 1 of the main text with $\omega_0 = 1/3, A = 1, l = 1, L = 3, \alpha = l/L, \beta = A/L, a = b = 0.2$, and $\varepsilon_x = \varepsilon_z = 2$ (for simplicity, we keep $\varepsilon_x = \varepsilon_z$, the anisotropy is already induced by the helix

along the x direction). In the effective media calculation, we set $\varepsilon_0 = \mu_0 = c = 1$, where c is the light velocity in vacuum.

Exactly accompanying with this effective Hamiltonian, the corresponding dispersive constitutive relation reads,

$$\mathbf{D} = \varepsilon \mathbf{E} + i\gamma \mathbf{H} \tag{5}$$

$$\mathbf{B} = \mu \mathbf{H} - i\gamma \mathbf{E} \tag{6}$$

where ε, μ, γ only have diagonal elements,

$$\varepsilon = \begin{bmatrix} \varepsilon_x + \dfrac{\alpha l}{\omega_0^2 - \omega^2} & 0 & 0 \\ 0 & 1 - \dfrac{\omega_p^2}{\omega^2} & 0 \\ 0 & 0 & \varepsilon_z \end{bmatrix} \tag{7}$$

$$\mu = \begin{bmatrix} 1 + \dfrac{\beta A \omega^2}{\omega_0^2 - \omega^2} & 0 & 0 \\ 0 & 1 & 0 \\ 0 & 0 & 1 \end{bmatrix} \tag{8}$$

$$\gamma = \begin{bmatrix} \dfrac{l \beta \omega}{\omega_0^2 - \omega^2} & 0 & 0 \\ 0 & 0 & 0 \\ 0 & 0 & 0 \end{bmatrix} \tag{9}$$

Supplementary References

1 Zhao, R., Koschny, T. & Soukoulis, C. M. Chiral metamaterials: retrieval of the effective parameters with and without substrate. *Optics Express* **18**, 14553-14567 (2010).

2 Raman, A. & Fan, S. Photonic Band Structure of Dispersive Metamaterials Formulated as a Hermitian Eigenvalue Problem. *Physical Review Letters* **104**, 087401 (2010).
